# Altered Cigarette Smoke-Induced Lung Inflammation Due to Ablation of Grx1

**DOI:** 10.1371/journal.pone.0038984

**Published:** 2012-06-18

**Authors:** Ine Kuipers, Ken R. Bracke, Guy G. Brusselle, Scott W. Aesif, Renske Krijgsman, Ilja C. Arts, Emiel F. M. Wouters, Niki L. Reynaert

**Affiliations:** 1 Department of Respiratory Medicine, Nutrim School for Nutrition, Toxicology and Metabolism, Maastricht University Medical Centre, Maastricht, The Netherlands; 2 Laboratory for Translational Research in Obstructive Pulmonary Diseases, Department of Respiratory Medicine, Ghent University Hospital, Ghent, Belgium; 3 Department of Pathology, George Washington University Hospital, Washington, D.C., United States of America; 4 Department of Epidemiology, Nutrim School for Nutrition, Toxicology and Metabolism Maastricht University Medical Centre, Maastricht, The Netherlands; University Hospital Freiburg, Germany

## Abstract

Glutaredoxins (Grx) are redox enzymes that remove glutathione bound to protein thiols, know as S-glutathionylation (PSSG). PSSG is a reservoir of GSH and can affect the function of proteins. It inhibits the NF-κB pathway and LPS aspiration in Grx1 KO mice with decreased inflammatory cytokine levels. In this study we investigated whether absence of Grx1 similarly repressed cigarette smoke-induced inflammation in an exposure model in mice. Cigarette smoke exposure for four weeks decreased lung PSSG levels, but increased PSSG in lavaged cells and lavage fluid (BALF). Grx1 KO mice had increased levels of PSSG in lung tissue, BALF and BAL cells in response to smoke compared to wt mice. Importantly, levels of multiple inflammatory mediators in the BALF were decreased in Grx1 KO animals following cigarette smoke exposure compared to wt mice, as were levels of neutrophils, dendritic cells and lymphocytes. On the other hand, macrophage numbers were higher in Grx1 KO mice in response to smoke. Although cigarette smoke *in vivo* caused inverse effects in inflammatory and resident cells with respect to PSSG, primary macrophages and epithelial cells cultured from Grx1 KO mice both produced less KC compared to cells isolated from WT mice after smoke extract exposure. In this manuscript, we provide evidence that Grx1 has an important role in regulating cigarette smoke-induced lung inflammation which seems to diverge from its effects on total PSSG. Secondly, these data expose the differential effect of cigarette smoke on PSSG in inflammatory versus resident lung cells.

## Introduction

The lung continuously encounters oxidants from inhalation and is therefore well equipped with a high concentration of the antioxidant glutathione (GSH). GSH acts as an electron donor and is used by glutathione peroxidase to reduce peroxides, resulting in oxidized glutathione (GSSG) [1]. Cigarette smoke is known to acutely deplete GSH, for instance by directly reacting with GSH to form non-reducible glutathione-aldehyde derivatives [2], thereby decreasing the lungs’ anti-oxidant capacity and making it vulnerable to oxidant-induced injury. On the other hand, as an adaptive response to oxidative stress, such as upon chronic smoking, levels of GSH increase in the epithelial lining fluid due to upregulation of the rate limiting enzyme in GSH synthesis, γ-glutamylcysteine ligase [3].

Besides being oxidized itself, glutathione can in conditions of mild oxidative stress, also bind to cysteine residues in proteins. This posttranslational modification is known as S-glutathionylation and protects proteins from irreversible oxidations. Glutaredoxins (Grx) or thioltransferases are redox enzymes that, under physiological conditions, can reverse S-glutathionylation. S-glutathionylation does not only protect the targeted protein thiol groups from further irreversible oxidations, but also has been shown to modulate protein function when the targeted cysteine residue is critical to its function [4]. Examples include mediators of cell death and inflammation such as procaspase-3 [5], multiple members of the NF-κB pathway (reviewed in [6]), and matrix metalloproteases [7]. Inhibition has been shown for caspase 3, as well as NF-κB, whereas MMP9 has been shown to be activated by this redox modification. Therefore glutaredoxins play an important role in redox-modulated protein function by regulating S-glutathionylation. Several mammalian Grxs have been identified. Grx1 localizes primarily to the cytosol and Grx2 is present in the mitochondria and nucleus [8].

In many pulmonary diseases, including COPD, the importance of glutathione homeostasis is described [3], whereas S-glutathionylation and Grxs have hardly been investigated. Grx1 expression in the lungs has been found to be predominantly localized in macrophages and bronchial epithelium. In a mouse model of allergic airway disease and after acute exposure to LPS Grx1 expression was increased [9], [10], [11]. In patients with COPD on the other hand, Grx1 was decreased and specifically the number of Grx1 positive macrophages was found to be positively correlated with lung function [10]. In line with these clinical findings, we have previously reported that cigarette smoke extract downregulated Grx1 levels, which was associated with increased protein S-glutathionylation in lung epithelial cells. Moreover, primary epithelial cells from Grx1 knock out mice were more prone to smoke-induced cell death and displayed higher levels of protein S-glutathionylation compared to controls [12]. I*n vivo* on the other hand, we found smoke exposure to decrease protein S-glutathionylation, while also decreasing Grx1 levels and total Grx activity [13].

Targeted S-glutathionylation is described to inhibit multiple members of the pro-inflammatory NF-κB pathway, including IKKα, IKKβ and Rel A [14], [15]. We have previously described that LPS exposure in the context of ablation of Grx1 failed to activate NF-κB and decreased inflammatory cytokine levels [11]. In the current study we set out to investigate whether absence of Grx1 similarly represses cigarette smoke-induced inflammation in a subacute exposure model in mice. Rather than focusing on individual NF-κB members, we investigated the differential inflammatory response of mouse lungs as well as primary epithelial cells and macrophages to cigarette smoke.

## Materials and Methods

### Mice and Primary Cell Culture

Male *Grx1^−/−^* mice, a kind gift of Dr. Ho (Wayne State University, Detroit, MI), and WT C57BL/6 controls (n = 10 per group) were exposed to cigarette smoke for four weeks as described previously [16]. Briefly, mice were exposed whole body to the tobacco smoke of 5 Reference Cigarettes 3R4F without filter (University of Kentucky, Lexington, KY) four times a day with 30 min smoke-free intervals, 5 days a week for 4 weeks. During the exposure an optimal smoke to air ratio of 1∶6 was obtained. The control groups were exposed to room air. Additional unexposed mice were used to isolate primary tracheal epithelial cells (MTE) as described previously [17] with minor modifications [18] and pulmonary macrophages by saline lavage. Cells were cultured in full medium lacking phenol red for 24 h prior to stimulation. The local ethics committee for animal experimentation of the faculty of medicine and health sciences of Ghent University, Belgium granted approval for all *in vivo* procedures under ECD07/04.

### Bronchoalveolar Lavage (BAL)

5 days after the last exposure mice were euthanized with an overdose of pentobarbital and a cannula was inserted into the trachea. Three times 300 µl HBSS, free of Ca^2+^ and Mg^2+^ and supplemented with 1% BSA, followed by 3 times 1 ml HBSS supplemented with 0.05 mM EDTA, was instilled through the cannula and recovered by gentle aspiration. All lavage fractions were pooled, centrifuged and the cell pellet washed twice and resuspended in 1 ml HBSS. Total and differential cell counts were performed in a Bürker chamber and cytocentrifuged preparations stained with May-Grünwald-Giemsa respectively. Flow cytometric analysis of BAL cells was performed as described previously to enumerate dendritic cells, macrophages, neutrophils and T-lymphocyte subsets [19].

### Lung Tissue Processing and Cell Counts

After rinsing of the pulmonary and systemic circulation, the left lung was used for histology by intratracheal infusion of 4% PFA and embedding in paraffin. Single cell suspensions were prepared from the right lung by mincing thoroughly, digesting and RBC lysis. Cell counts were performed with a Beckman Coulter counter and flow cytometric analysis was performed as described previously to enumerate dendritic cells, macrophages, neutrophils and T-lymphocyte subsets [19]. The other part of the right lung was snap frozen in liquid nitrogen for biochemical assessments.

### Cigarette Smoke Extract

3R4F Research Cigarettes, from the University of Kentucky (Lexington, KT, USA), were removed from their filters and cigarette smoke extract (CSE) was made fresh before every experiment according to [20].

### Quantitative Determination of S-Glutathionylated Proteins Using 5,5′-dithio-bis(2-Nitrobenzoic Acid) (dTNB)

200 µl of BAL fluid or 200 µg of lung protein homogenate was acetone precipitated for 20 minutes at −20°C and spun down for 5 minutes at 3000×g. Pellets were next resuspended and sonicated in 200 µl of ice-cold extraction buffer containing 0.2% Triton-X 100 and 0.6% sulfosalicyclic acid in 0.1 M potassium phosphate buffer with 5 mM EDTA disodium salt (KPE), pH 7.5. After 2 freeze-thaw cycles, samples were centrifuged at 3000×g for 4 min at 4°C. To remove glutathione (GSH) from proteins, the pellet was treated with 100 µl of 1% NaBH_4_ in water and neutralized with 40 µl of 30% metaphosphoric acid. Samples were centrifuged at 1000×g for 15 min and the supernatant was used to determine the GSH content using the dTNB GSSG reductase recycling method [21]. 20 µl of KPE, GSH standards and samples were pipetted into a 96-well microtiter plate and freshly prepared, equal volumes of dTNB and GSSG reductase were added in the dark. After 30 seconds, β-NADPH was added to start the conversion of dTNB to TNB and the absorbance at 412 nm was read every 30 seconds for 2 minutes. A standard curve was performed using a concentration range of GSH. NaBH_4_ was omitted for each sample, as a negative control. Values were corrected for protein content and data are expressed as nmol GSH per milligram of protein.

### Grx1 Catalyzed Cysteine Derivatization for In Situ Detection of S-glutathionylated Proteins

Frozen cytospins were thawed and washed twice with PBS before being fixed with 4% paraformaldehyde (PFA) for 10 minutes at RT. After three washes with PBS slides were permeabilized and free thiol groups were blocked using a buffer containing 25 mmol/L 4-(2-hydroxyethyl)-1-piperazineethanesulfonic acid, pH 7.4, 0.1 mmol/L EDTA, pH 8.0, 0.01 mmol/L neocuproine, 40 mmol/L *N*-ethylmaleimide (Sigma) and 1% Triton (Sigma) for 30 minutes to an hour. After three washes with PBS, S-glutathionylated cysteine groups were reduced by incubation with 13.5 µg/ml human Grx1 (Lab Frontiers), 35 µg/ml GSSG reductase (Roche), 1 mmol/L GSH (Sigma), 1 mmol/L NADPH (Sigma), 18 µmol EDTA and 137 mmol/L Tris · HCl, pH 8.0, for 20 minutes. As a control GSH was left out of this mix. After three washes with PBS, newly reduced cysteine residues were labelled with 1 mmol/L *N*-(3-maleimidylpropionyl) biocytin (MPB) (Roche) for 1 hour, after which excess MPB was removed by three washes with PBS. Next, cells were incubated with 0.5 µg/ml streptavidin-conjugated Alexa Fluor 568 for 30 minutes. Nuclei were stained using 0.5 µg/ml DAPI Blue. Cells were then mounted, coverslipped and analyzed by fluorescent microscopy using a Nikon Eclipse E800 microscope. All conditions were scanned using identical instrument settings that did not result in saturation of pixel intensities. Semi-quanititative assessment of the staining intensity was conducted by dividing mean red fluorescence intensity (PSSG staining) by the mean blue fluorescence intensity (nuclear DAPI staining) using Image J software. Mean relative fluorescence intensity (RFI) values and SEM were thus obtained.

### Grx1 Staining

Macrophages were fixed with 4% PFA for 10 min at RT. After permeabilization and blocking non-specific binding sites using 0.1%triton, 1% BSA in PBS, primary antibody against Grx1 (Imco) was incubated for 1 h followed by Alexa fluor 488 labelled secondary anti-goat antibody for 1 h. Nuclei were counterstained with DAPI and cells were coverslipped. Semi-quanititative assessment of the staining intensity was conducted by dividing mean green fluorescence intensity (Grx1 staining) by the mean blue fluorescence intenstiy (nuclear DAPI staining) using Image J software. Mean relative fluoresence intensity (RFI) values and SEM were thus obtained.

### Multiplex for Cytokine Measurement

To quantify concentrations of 23 cytokines and chemokines in BALF we used a Bio-Plex mouse cytokine 23-plex Panel (IL-1a, IL-1β, IL-2, IL-3, IL-4, IL-5. IL-6, Kc, IL-9, IL-10, IL-12(p40), IL-12(p70), IL-13, IL-17, Eotaxin, G-CSF, GM-CSF, IFN- γ, MCP-1(MCAF), MIP-1α, MIP-1β, RANTES and TNF-α). Assays were performed as described by the manufacturer’s instructions. The analysis was done with a Luminex 100 IS 2.3 system using the Bio-Plex Manager 4.1.1. software.

### Kc ELISA

Kc levels in cell culture medium were measured using a commercially available ELISA kit (R&D systems, Inc. Minneapolis, USA) according to the manufacturer’s instructions.

### QPCR

Total RNA was isolated from lungs or cells using the RNeasy Mini kit (QIAGEN, California, USA) and an equal amount was reverse transcribed into cDNA using the Reverse-iT 1st strand Synthesis Kit (Abgene, Epsom, UK). Primers for human HPRT (FW:AGAATGTCTTGATTGTGGAAGA; REV:ACCTTGACCATCTTTGGATTA), Grx1 (FW:TTTACAACAGCTCACCGGAG; REV:TCACTGCATCCGCCTATG) and Kc (Fw: CACTGCACCCAAACCGAAG; REV: TCAGGGTCAAGGCAAGCC) were used. PCR reactions were performed on an *iCycler iQ* Real-Time PCR system (BioRad, Hercules, California, USA) using the SYBRgreen dye (BioRad). Relative mRNA expression of genes was calculated using the standard curve method.

### Statistical Analyses

Between-group comparisons were analyzed using the Kruskal-Wallis test, followed by Mann-Whitney *U* test (SPSS 17). Unless indicated otherwise, data are expressed as mean and standard deviation. A *p*-value <0.05 was considered statistically significant.

## Results

### BAL Fluid Cell Counts and Differentials in Wild Type Versus Grx1 KO Mice Exposed to Air and Smoke

**Figure 1:**
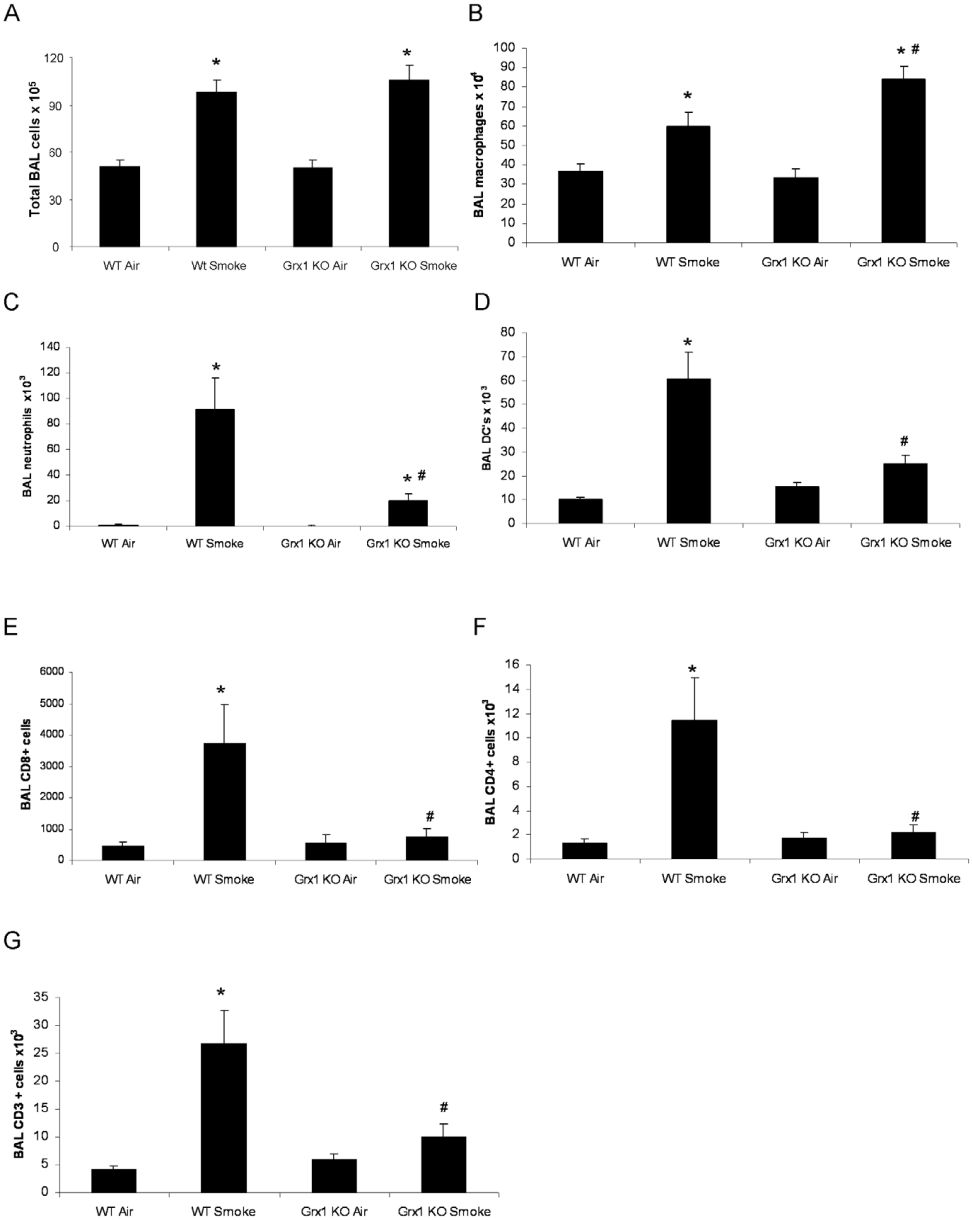
BAL fluid cell counts and differentials in wild type versus Grx1 KO mice exposed to air and smoke. Total BAL fluid cells (A), total numbers of macrophages (B), neutrophils (C), dendritic cells (D), CD3+ cells (F), CD4+ cells (G) and CD8+ cells (H) in BAL fluid represented as mean ± SD. * represents p<0.05 between air and smoke exposed mice, # represents p<0.05 between WT smoke and KO smoke.

When analyzing lavaged cells, the total number of BALF cells was found to be significantly elevated in wild type and Grx1 KO mice due to cigarette smoke compared to respective air exposed controls. The level of increase in total cell numbers in the BALF did not differ between the two mouse strains (Figure 1A). Macrophage cell counts in BALF also increased with cigarette smoke compared to respective air exposed controls, but significantly more so in Grx1 KO mice than in WT (Figure 1B). The smoke-induced increase in the number of neutrophils, dendritic cells, CD8+, CD4+ and CD3+ cells on the other hand was significantly dampened in Grx1 KO compared to WT mice (Figure 1 C–G).

### Lung Tissue Differential Cell Counts in Wild Type versus Grx1 KO Mice Exposed to Air or Smoke

**Figure 2:**
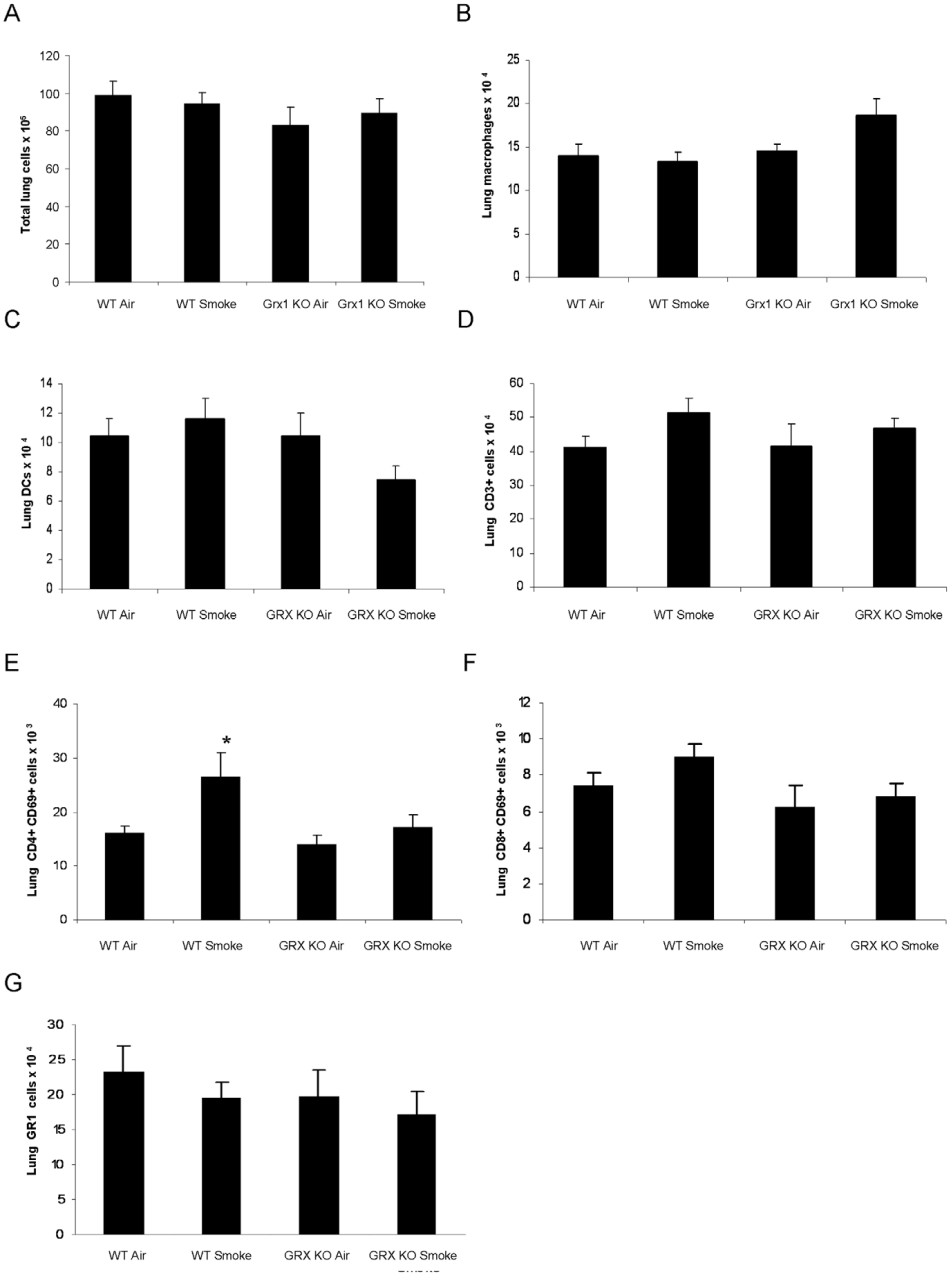
Lung tissue differential cell counts in wild type versus Grx1 KO mice exposed to air or smoke. Total lung cells (A), total numbers of lung macrophages (B), dendritic cells (C), CD3+ cells (D), CD4+ CD69+ cells (E) CD8+ CD69+ cells (F) and GR1 cells (G), represented as mean ± SD. * represents p<0.05 between air and smoke exposed mice.

Next, we investigated total and differential cell numbers in lung tissue of the wild type and Grx1 KO mice. Figure 2A shows that there is no significant difference in total cell numbers of the lung tissue between the Grx1 KO and wild type mice, under basal conditions and after exposure to cigarette smoke.

Although the numbers of macrophages tended to be higher in lung tissue of Grx1 KO mice compared to wild type controls, this was not statistically significant (Figure 2B). In addition, the numbers of lung dendritic cells (Figure 2C), lung CD3+ cells (Figure 2D), lung CD8+CD69+ (Figure 2F) and GR1+ cells (Figure 2G) were not affected by smoke exposure in the WT animals, or in the Grx1 KO mice. The numbers of lung CD4+CD69+ cells on the other hand was significantly increased in wild type mice exposed to cigarette smoke. This increase was however not present in the Grx1 KO mice after cigarette smoke exposure (Figure 2E).

### BAL Fluid Cytokines in Wild Type Versus Grx1 KO Mice Exposed to Air and Cigarette Smoke

**Figure 3:**
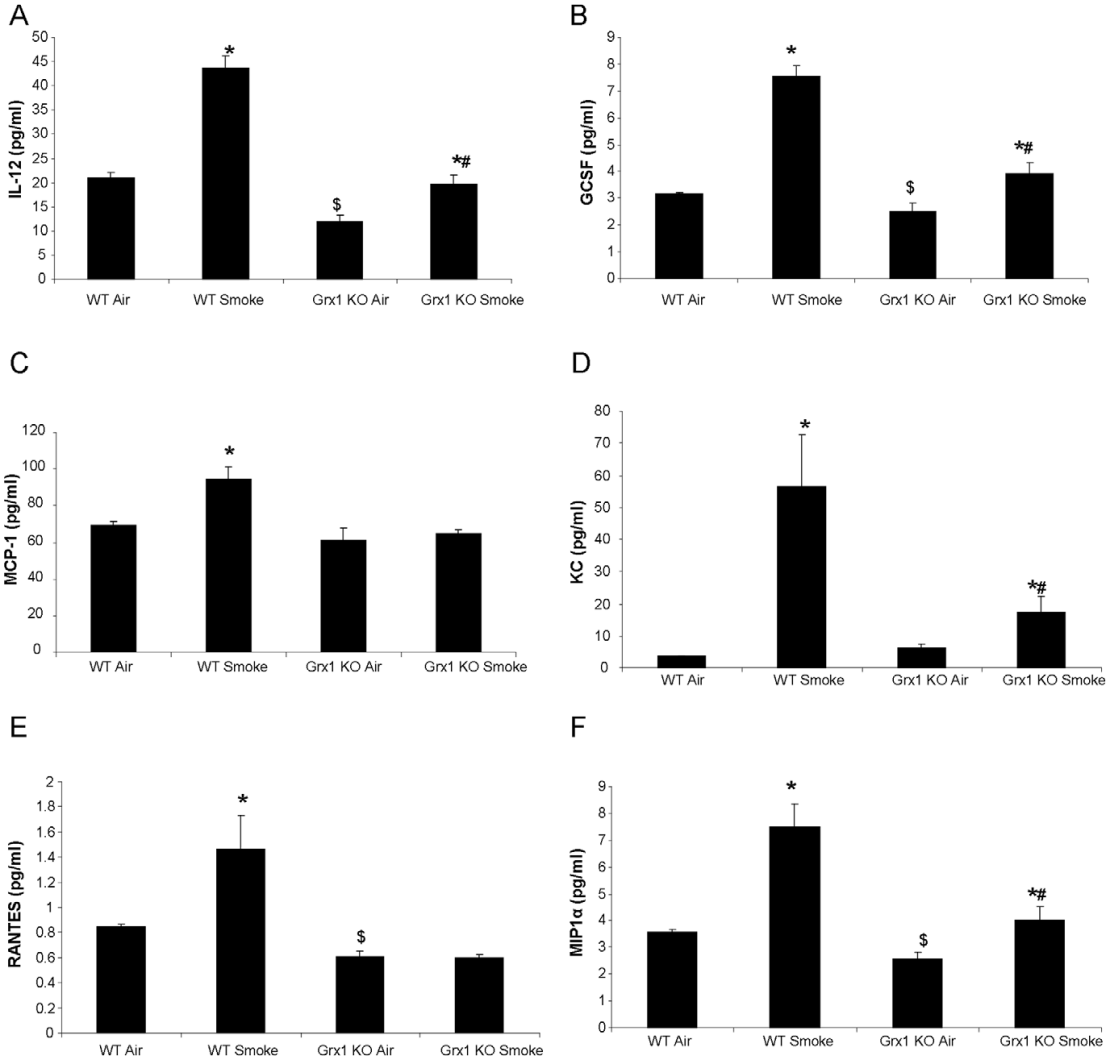
BAL fluid cytokines in wild type versus Grx1 KO mice exposed to air and cigarette smoke. Measurement of cytokines in the BAL fluid by multiplex: IL 12 (A), GCSF (B), MCP1 (C), KC (D), RANTES (E) and MIP1α (F) expressed in pg/ml and represented as mean ± SD. * represents p<0.05 between air and smoke exposed mice, # represents p<0.05 between WT smoke and KO smoke and $ represents p<0.05 between WT air and Grx1 KO air.

We next assessed a broad panel of inflammatory mediators in BALF in order to determine a cause for the diminished influx of inflammatory cells into the lungs of Grx1 mice after smoke exposure. While analyzing the inflammatory mediators measured in the BAL fluid of wild type versus Grx1 KO mice, the general observation was that these are decreased in Grx1 KO mice after exposure to cigarette smoke compared to wild type controls exposed to cigarette smoke (Figure 3). Specifically, cigarette smoke exposure significantly increased the BALF concentration of IL12(p40), GCSF, MCP-1, KC, RANTES and MIP-1alpha in wild type mice. In the Grx1 KO, the cigarette smoke-induced upregulation of these cytokines was significantly impaired, compared to the wild type controls. Moreover, the baseline levels of IL12(p40), GCSF, RANTES, MIP-1α, TNFα (data not shown) and IFNγ (data not shown) were found to be lower in the BAL fluid of Grx1 KO mice compared to wild type mice.

### S-glutathionylation in Lungs and BAL Fluid Cells of Grx1 KO Compared to Wild Type Mice Exposed to Cigarette Smoke and Air

**Figure 4:**
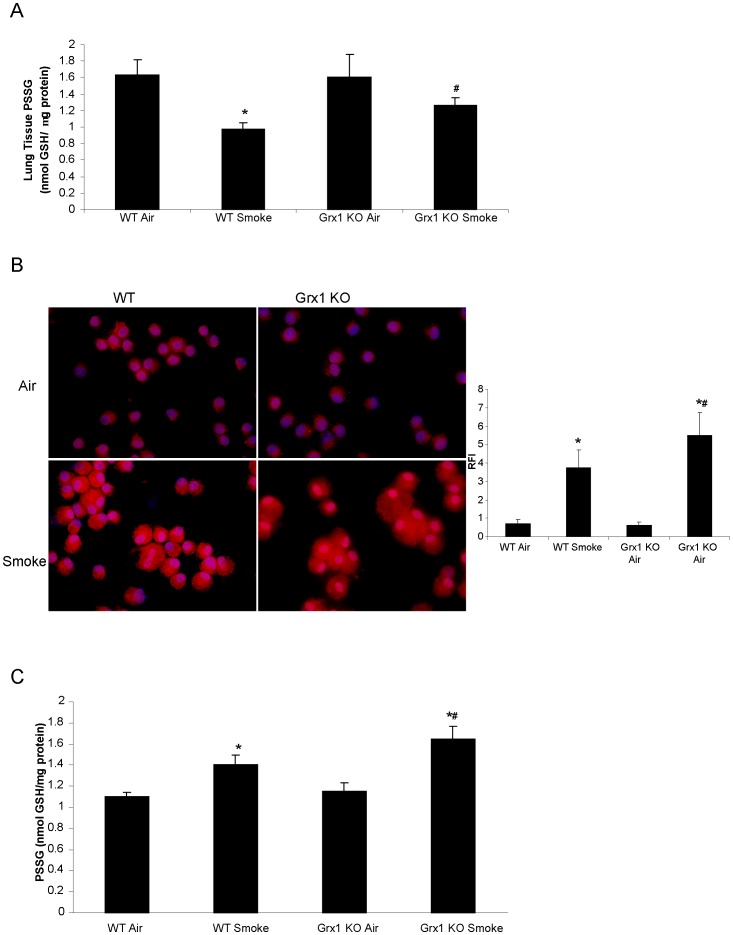
S-glutathionylation in lungs and BAL fluid cells and BAL fluid of Grx1 KO compared to wild type mice exposed to cigarette smoke and air. S-glutathionylation of proteins quantified by the DTNB assay in lung tissue of Grx1 KO compared to wild type mice exposed to cigarette smoke and air, data shown in nmol GSH/mg protein and represented as mean ± SD (A). S-glutathionylation of proteins visualised by the biotin switch staining in BAL fluid cells of Grx1 KO compared to wild type mice exposed to cigarette smoke and air, the outer right panel shows the quantification of staining expressed as relative fluorescent intensity of red staining (S-glutathionylated proteins) compared to blue, nuclear DAPI staining and represented as mean ± SD (B). S-glutathionylation of proteins quantified by the DTNB assay in BAL fluid of Grx1 KO compared to wild type mice exposed to cigarette smoke and air, data shown in nmol GSH/µg protein and represented as mean ± SD (C). * represents p<0.05 between air and smoke exposed mice, # represents p<0.05 between WT smoke and KO smoke.

Since the major function of Grx1 is to catalyze deglutathionylation under physiological conditions, we next investigated the levels of total protein S-glutathionylation in lung tissue. As demonstrated in Figure 4A, exposure to cigarette smoke for four weeks lead to a significant decrease in S-glutathionylation of proteins in the lung tissue of wild type mice as reported previously. In the Grx1 KO mice, the smoke-induced decrease in protein S-glutathionylation did not reach statistical significance, but the levels observed after smoke exposure were significantly elevated compared to those in the wild type mice exposed to smoke. The levels of free GSH were decreased after smoke exposure as well, but no differences were observed between Grx1 KO and wild type mice (data not shown).

When previously visualizing protein S-glutathionylation in whole lung tissue, we noted that this was not decreased in inflammatory cells after smoke exposure [13]. We therefore analyzed protein S-glutathionylation in cells obtained by BAL using the biotin-switch approach and found that protein S-glutathionylation in these BAL fluid cells of mice exposed to smoke was increased compared to BAL fluid cells from air exposed mice. Moreover, S-glutathionylation in BAL fluid cells of Grx1 KO mice was higher after cigarette smoke exposure than in the wild type controls (Figure 4B–C). As expected, most of the cells in the cytospins had the morphological characteristics of macrophages. In cell free BALF, similar trends were observed with increased protein S-glutathionylation after smoke exposure and heightened levels in Grx1 KO mice compared to wt controls (Fig. 4D). At baseline, no differences were observed between WT and Grx1 KO mice.

### Grx Expression in Lung Tissue and Macrophages of Mice Exposed to Cigarette Smoke

**Figure 5:**
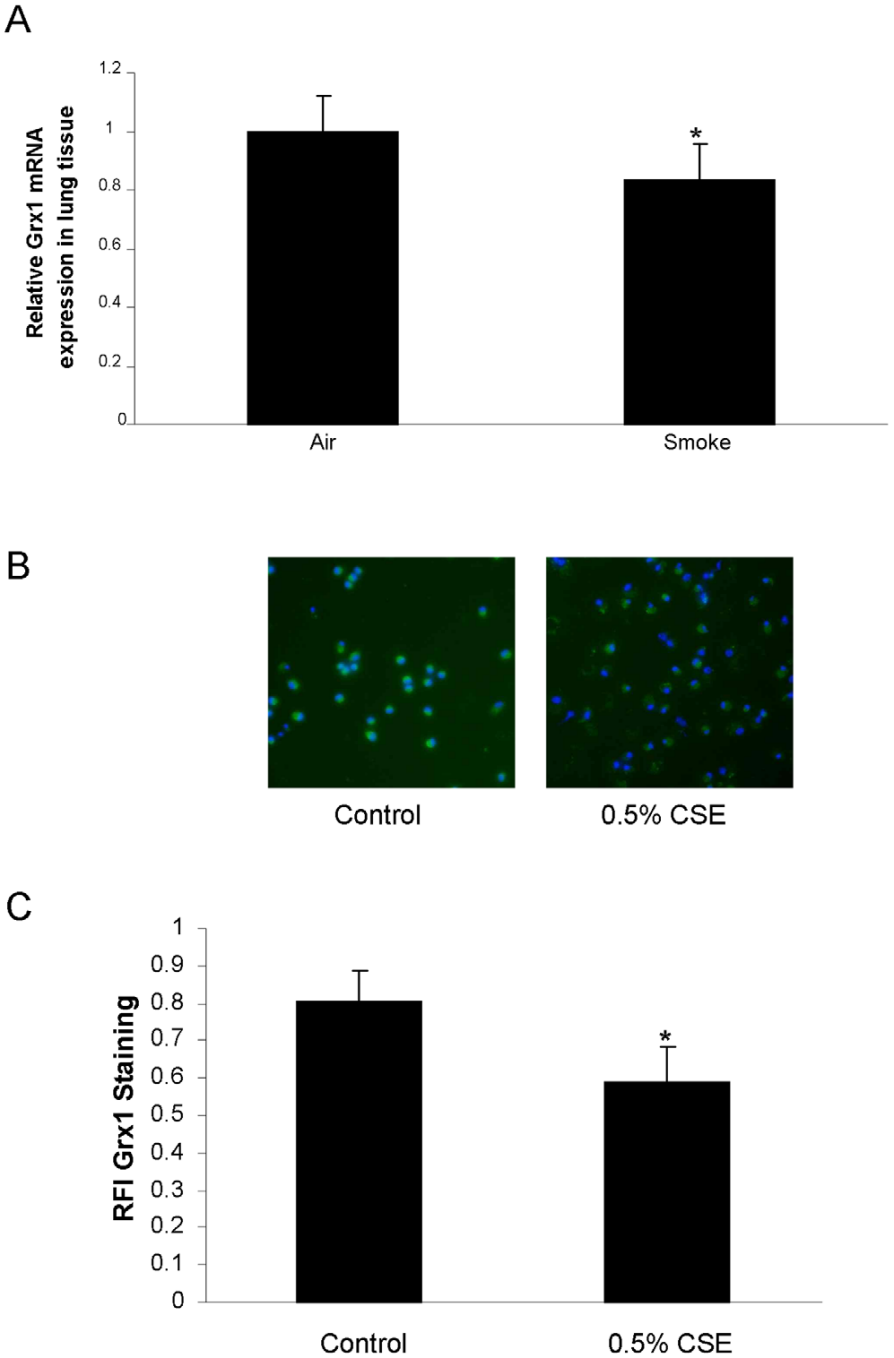
Grx1 expression in lung tissue and primary macrophages of mice exposed to cigarette smoke. (A) Grx1 mRNA expression corrected for HPRT mRNA expression in lung tissue of mice exposed to air and cigarette smoke, represented as mean ± SD. (B) Fluorescent Grx1 staining in primary macrophages after 24 hours of control or 0.5% cigarette smoke extract exposure. The green staining represents Grx1 protein expression, whereas blue represents the nuclear DAPI staining. Quantification of fluorescent Grx1 staining is expressed as RFI in (C).

We have previously shown that exposure of pulmonary epithelial cells to cigarette smoke extract (CSE) leads to decreased expression of Grx1 mRNA and protein. In Figure 5A we first confirmed these data in lung tissue of smoke exposed mice. Indeed, Grx1, but not Grx2 (data not shown) mRNA levels are significantly decreased after four weeks of cigarette smoke exposure compared to air exposed controls in wild type mice. Given the differential response of structural and inflammatory cells to smoke with respect to protein S-glutathionylation, we also assessed the effects of CSE on Grx expression in primary macrophages isolated from mice by fluorescent staining for Grx1 (represented in figure 5B and C) and confirm that smoke exposure also represses Grx1 protein levels in these cells.

### KC in Primary Macrophages Versus Primary Epithelial Cells from Wild Type and Grx1 KO Mice after Exposure to Cigarette Smoke Extract

**Figure 6:**
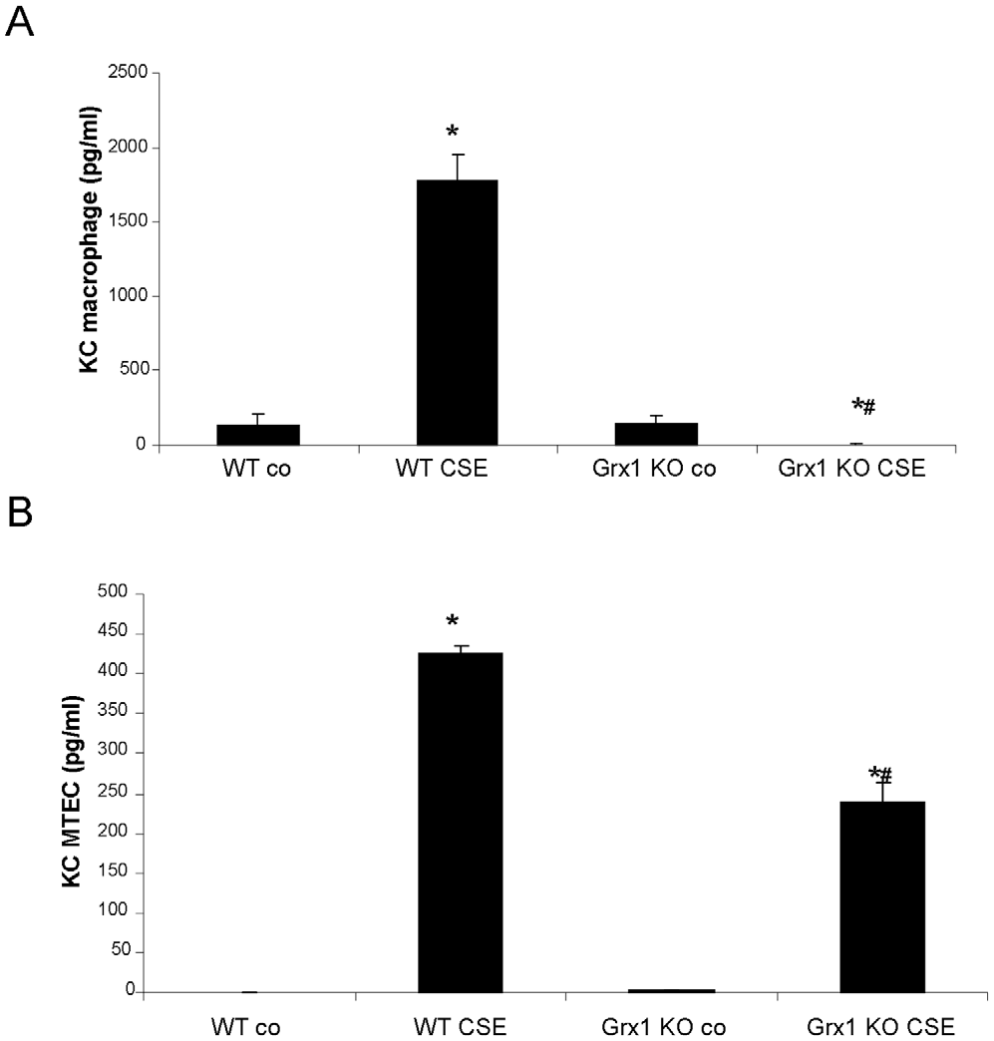
KC in primary macrophages versus primary epithelial cells from wild type and Grx1 KO mice after cigarette smoke extract exposure. KC in medium of primary macrophages (A) and of mouse tracheal epithelial cells (B) from wild type and Grx1 KO mice exposed to air or 0.1% cigarette smoke extract for 24 hours expressed in pg/ml.

Protein S-glutathionylation appears thus to be differentially regulated in structural and inflammatory cells after smoke exposure, albeit independently from Grx1. We therefore asked whether this would differentially impact inflammatory mediator production in these cell types in response to cigarette smoke, given that we have previously shown that the Grx1-PSSG axis is important in determining the extent of NF-κB activation and the levels of cytokines and chemokines such as KC in response to pro-inflammatory stimuli. When performing an ELISA for KC on culture supernatants of macrophages isolated from wild type mice, we found a significant increase in KC after exposure to CSE. In contrast, the Grx1 KO macrophages displayed decreased KC concentrations in their medium after exposure to CSE (Figure 6A). Also in MTECs the production of this chemokine was increased by cigarette smoke from both WT and Grx1 KO mice. Moreover, this response to CSE was significantly decreased in the Grx1 KO MTECs compared to the cells from wild types (Figure 6B).

## Discussion

It is becoming increasingly clear that GSH and its associated enzymes and the redox state of cells in general do not only play a role in protection against oxidative stress and damage, but also determine the outcome of discrete receptor-induced ROS-mediated signaling events involved in immune responses. Specifically shown here is that Grx1 and PSSG are key modulators of the *in vivo* response to cigarette smoke. Although there was no difference in the total amount of BAL fluid and lung cells between wild type and Grx1 KO mice after smoke exposure, the pattern of inflammatory cells found in the BAL fluid was different. The Grx1 KO mice actually accumulate macrophages in the BAL fluid after 4 weeks of cigarette smoke exposure, while in contrast to the wild type mice the increase of neutrophils, dendritic cells and CD3+, CD4+ and CD8+ T-cells was significantly impaired. This altered pattern of inflammatory cells in the BALF was mirrored by significant differences in the levels of chemokines and cytokines. After four weeks of cigarette smoke exposure the BALF of Grx1 KO mice contained significantly less KC, RANTES, MCP-1, IL12, GCSF and MIP1α compared to the wild type controls exposed to the same amount and duration of cigarette smoke. The decreased levels of inflammatory mediators in the BAL fluid of Grx1 KO mice can be caused by the diminished levels of inflammatory cells other than macrophages. On the other hand, it could be attributed to the immaturity of these macrophages as we reported previously that alveolar macrophages isolated from Grx1 KO mice are smaller, express lower levels of the hematopoietic cell specific transcription factor PU.1 and displayed decreased phagocytosis capacity *in vitro* compared to alveolar macrophages from wild type animals [11]. Also, LPS-induced NF-κB activation and inflammatory mediator production were found to be attenuated in the Grx1 KO macrophages as well as primary tracheal epithelial cells.

The Grx1 KO mice have been used by others to investigate the role of Grx1 in cigarette smoke induced lung inflammation. This study however employed a three day smoke exposure protocol and showed a much different outcome compared to the results obtained here. After a three day exposure regimen, the number of neutrophils were reported to be elevated in the Grx1 KO mice, in conjunction with increased levels of KC and MCP-1 compared to wild type controls [15]. The main difference between studies is the length of exposure and might indicate that the role of Grx1 is opposite in an acute versus a more chronic exposure to cigarette smoke. This is also supported by the early increase in KC levels observed after LPS exposure in the Grx1 KO mice, compared to the attenuation of other mediators at more protracted time points [11]. Not only the role of Grx1 in smoke-induced inflammation was found to be different, but in our model smoke exposure was found to decrease protein S-glutathionylation compared to the increase observed in the acute three day model of cigarette smoke exposure. We did not observe a difference in basal level of protein S-glutathionylation between WT and Grx1 KO mice, but smoke failed to affect PSSG in Grx1 KO mice. The level of PSSG in the lungs of Grx1 KO mice was significantly higher after smoke compared to the wild type animals. The differential response in S-glutathionylation of proteins in the two models might thus be involved in the discrepancies found in lung inflammation between acute and chronic smoke exposure. It has been reported by our laboratory that upon oxidative stress, targeted S-glutathionylation of IKKβ inhibits its activity and thus inhibits Rel A nuclear translocation. Binding of NF-κB to its consensus sequence has also been shown to be negatively affected by S-glutathionylation [14]. Secondly, we have previously shown that Grx1 KO mice and primary epithelial cells isolated from these mice, show a markedly diminished response to LPS with respect to NF-κB activation and inflammatory mediator production [11]. Since the Grx1 KO mice have more overall S-glutathionylation in the lung and in the BAL cells after smoke exposure, this might, through the inhibition of nuclear Rel A translocation, contribute to the decreased inflammatory cytokine production observed in the Grx1 KO mice after four weeks of cigarette smoke exposure, compared to wild type mice. So it is likely that the differential response in PSSG levels in the acute versus the more chronic cigarette smoke exposure causes the difference in phenotype in the mouse lung.

When we assessed PSSG in lavaged cells and fluid from smoke compared to air exposed mice, we found increased S-glutathionylation of proteins in contrast to the observed decrease in whole lung tissue. In lung tissue, S-glutathionylation was decreased despite a decrease in Grx1 mRNA expression. When we isolated primary macrophages from mice and exposed them to cigarette smoke extract, there was a decrease in Grx1 protein levels. These findings in macrophages are in line with our previously published data showing decreased Grx1 levels in various pulmonary epithelial cells after smoke exposure [12]. Together these data indicate that overall protein S-glutathionylation can alter independently of differences in Grx1 and that the patterns of S-glutathionylation depend on cell type as well as stimuli and duration of stimulation.

It should be noted that the alteration in the overall protein S-glutathionylation pattern does not mean that all individually targeted proteins would be affected in the same direction. Although we did not investigate individual targets, in figure 6 of this manuscript we demonstrate that a differential effect on overall S-glutathionylation of proteins in response to smoke observed in different cell types, can still result in the same outcome. In particular, we demonstrate that smoke-induced KC production is decreased in both tracheal epithelial cells and macrophages isolated from Grx1 KO mice, irrespective of the effect on total protein S-glutathionylation.

Taken together, we demonstrate in this manuscript that by using a knock out mouse model for glutaredoxin 1, this redox mediating enzyme has an important role in regulating cigarette smoke induced lung inflammation.
